# Integrative Analysis of miRNA and mRNA Profiles in Response to Ethylene in Rose Petals during Flower Opening

**DOI:** 10.1371/journal.pone.0064290

**Published:** 2013-05-16

**Authors:** Haixia Pei, Nan Ma, Jiwei Chen, Yi Zheng, Ji Tian, Jing Li, Shuai Zhang, Zhangjun Fei, Junping Gao

**Affiliations:** 1 Department of Ornamental Horticulture, China Agricultural University, Beijing, China; 2 Boyce Thompson Institute for Plant Research, Cornell University, Ithaca, New York, United States of America; 3 USDA Robert W. Holley Center for Agriculture and Health, Ithaca, New York, United States of America; Ecole Normale Superieure, France

## Abstract

MicroRNAs play an important role in plant development and plant responses to various biotic and abiotic stimuli. As one of the most important ornamental crops, rose (*Rosa hybrida*) possesses several specific morphological and physiological features, including recurrent flowering, highly divergent flower shapes, colors and volatiles. Ethylene plays an important role in regulating petal cell expansion during rose flower opening. Here, we report the population and expression profiles of miRNAs in rose petals during flower opening and in response to ethylene based on high throughput sequencing. We identified a total of 33 conserved miRNAs, as well as 47 putative novel miRNAs were identified from rose petals. The conserved and novel targets to those miRNAs were predicted using the rose floral transcriptome database. Expression profiling revealed that expression of 28 known (84.8% of known miRNAs) and 39 novel (83.0% of novel miRNAs) miRNAs was substantially changed in rose petals during the earlier opening period. We also found that 28 known and 22 novel miRNAs showed expression changes in response to ethylene treatment. Furthermore, we performed integrative analysis of expression profiles of miRNAs and their targets. We found that ethylene-caused expression changes of five miRNAs (*miR156*, *miR164*, *miR166*, *miR5139* and *rhy-miRC1*) were inversely correlated to those of their seven target genes. These results indicate that these miRNA/target modules might be regulated by ethylene and were involved in ethylene-regulated petal growth.

## Introduction

MicroRNAs (miRNAs) are 20–24 nucleotide (nt)-long non-coding RNA species that play profound roles in plant development and in plant responses to abiotic and biotic stimuli by regulating expression of their target genes, mainly at the post-transcriptional level. In plants, most miRNA genes are transcribed by Pol II to primary miRNAs (pri-miRNAs) which are partially self-complementary and possess the fold-back hairpin structure [1]. The pri-miRNAs are then processed to generate precursor miRNAs (pre-miRNAs) by a protein complex consisting of the Dicer-like 1 (DCL1), the C2H2-zinc finger protein SERRATE 11 (SE), and the double-stranded RNA-binding protein HYPONASTIC LEAVES1 (HYL1) [1]. Mature miRNA duplex (miRNA/miRNA*) is excised from pre-miRNAs by DCL1 and each strand is methylated by HEN1 protein. The miRNA strand is ultimately loaded into the Argonaute (AGO) protein of RNA-induced silencing complex (RISC) to carry out its function [1], [2]. Mature miRNAs are able to regulate their target genes through at least four mechanisms: 1) direct cleavage of the target mRNAs, 2) translational inhibition of the targets, 3) regulation of the targets through secondary siRNAs, and 4) sequestration of the miRNA and targets through target mimicry [3]. Although translational repression of targets is the most important way for miRNA-mediated regulation in animals, in plants cleavage of the targets is predominant [1], [3].

Bioinformatic analysis shows that 21 miRNA families are likely well conserved in angiosperms, including *miR156*, *miR159*, *miR160*, *miR162*, *miR164*, *miR166*, *miR167*, *miR168*, *miR169*, *miR171*, *miR172*, *miR319*, *miR390*, *miR393*, *miR394*, *miR395*, *miR396*, *miR397*, *miR398*, *miR399* and *miR408*. Plants contain much more non-conserved miRNAs than conserved ones, for example, at least 48 non-conserved miRNAs have been found in *Arabidopsis thaliana* [4]. Recently, advances in high throughput DNA sequencing technology have enabled rapid and deeper discovery of non-conserved miRNAs from divergent plant species, including grape [5], barley [6], cucumber [7], olive [8], tomato [9], apple [10], and peach [11]. Currently, at least 4,677 mature miRNAs were identified from plants [12]. Moreover, many small RNA libraries were constructed from different plants subjected to hormonal and environmental treatments to identify novel and specific miRNAs in response to these stimuli. Resultantly, hundreds of miRNAs were found to be modulated by various hormones and stresses, including ABA, GA, auxin, pathogen, high-salinity, drought, cold, heat, mechanical stress, hypoxia and oxidative stresses [13]–[15].

*Rosaceae* is an economically important plant family that includes several important fruits and ornamental plants, such as apple, peach, strawberry and rose. Genome sequences of apple, peach and strawberry have been generated; however, research in small RNAs of the *Rosaceae* plant is still limited. Recently, miRNAs from apple and peach were reported [10], [11], [16], [17]. Unlike apple and peach which are important for their fresh fruits, rose is important for its beautiful and fragrant flowers. In the past century, rose has been the most important crop in the floriculture industry worldwide and cut roses account for approximately one third of cut flower trade in Europe [18]. In addition, rose possesses some unique morphological and physiological features, including recurrent flowering and highly divergent flower shapes, colors and volatiles, which are unable to be studied in other model plant systems, like *Arabidopsis thaliana* and tobacco [19].

Gaseous phytohormone ethylene is a crucial modulator in multiple biological processes, including seed germination, organ elongation, flowering, fruit ripening, organ senescence and abscission, as well as abiotic and biotic stress responses [20], [21]. It has been well known that ethylene can cause severe deterioration of flower quality in cut roses, mainly through the inhibition of petal expansion and acceleration of opening and senescence processes [22]. Although it has been extensively documented concerning the regulatory pattern of ethylene biosynthesis and signaling during flower opening and senescence in roses [23]–[27], the gene network downstream to ethylene signaling remains largely unknown. A recent study reported the identification of miRNAs in three modern rose cultivars and *Rosa rugosa*, and suggested that miRNAs could be involved in regulating genes related to coloring, like flavonoid biosynthetic genes [28]. However, the expression pattern of miRNAs in rose petals during flower opening and in response to ethylene still remains unexplored.

Here, we report small RNA profiling in rose petals during the rapid opening period and in response to ethylene treatment through high-throughput sequencing. In addition, we performed integral analysis of expression profiles of miRNAs and their predicted targets to further screen the bona fide miRNA-mRNA modules and discuss the possible biological roles of miRNAs differentially expressed in petals during flower opening and in response to ethylene.

## Results

### Construction and Sequencing of Small RNA Libraries from Rose Petals

The flower opening process is divided into seven stages in rose. The duration from unopened buds (stage 0) to partially opened flowers (Stage 3) is the rapid growth (RG) period [25], [29] and is important for the establishment of flower opening quality, especially the flower shape. Treatment with ethylene in the RG period can accelerate flower opening, but inhibit petal enlargement and even result in abnormal flower shapes [22], [27], [30].

To obtain a comprehensive survey of miRNAs in rose petals in the RG period and in response to ethylene treatment, we constructed and sequenced small RNA libraries from petals of unopened buds (stage 0, S0), opened buds (stage 2, S2), partially opened flowers (stage 2 flowers exposed to air for 24 h, C24), and ethylene-treated flowers (stage 2 flowers treated with 10 ppm ethylene for 24 h, E24).

**Figure 1:**
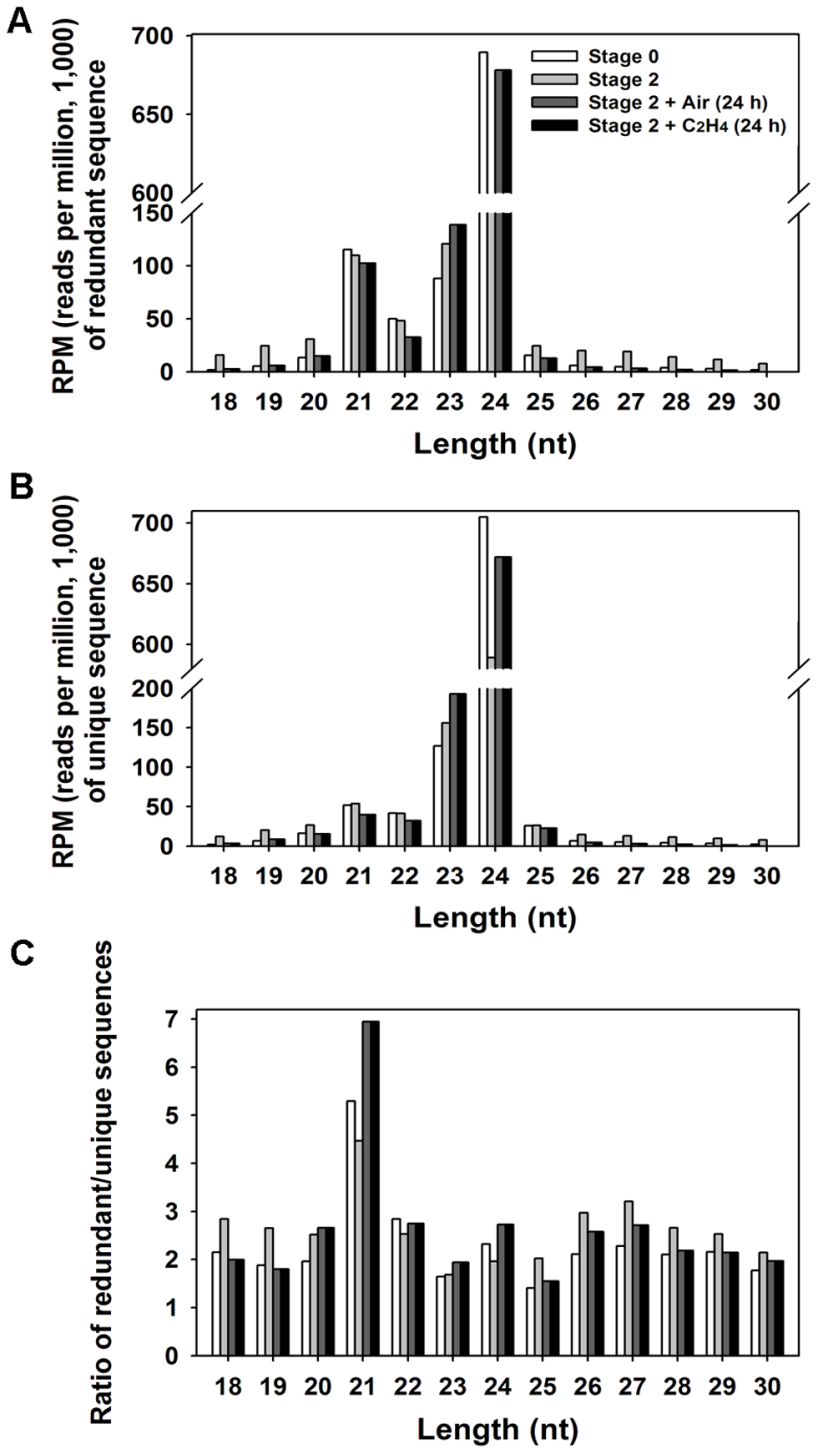
Size distribution of small RNA (sRNA) sequences from rose petals. The number of redundant (A) and unique (B) sequences from rose petals. (C) Redundancy ratio for sRNAs from rose petals.

We obtained 16,648,213, 6,069,761, 11,579,864, and 15,937,871 redundant reads of 10–40 nt from petals of S0, S2, C24, and E24 samples, respectively, after removing adaptors, low quality reads and contaminants (Table S1). The length distribution of the small RNAs ranged from 18 to 30 nt was examined and shown in Figure 1. In all four samples, 21-nt, 23-nt and 24-nt small RNAs were the major population, consistent with the size of Dicer-like protein cleavage products, and 24-nt was the most dominant, similar to the results obtained from most tested plants, such as *Arabidopsis thaliana*, rice, tomato, cucumber, apple and peach [7], [9], [10], [11], [16], [17], [31], [32]. The redundancy level of sRNAs was low for the 23-nt and 24-nt sRNAs, while the 21-nt sRNAs have the highest redundancy level, especially in the E24 library. This is different from that of cucumber, as the redundancy level of 22-nt sRNAs was reported to be the highest [7]. All the small RNA sequences have been deposited into NCBI SRA database under accession SRA066431.

### Identification of Conserved miRNAs in Rose Petals

It has been well known that miRNAs play critical roles in plant development and in plant responses to various abitotic and biotic stimuli [1]–[3]. To date, hundreds of plant miRNAs have been identified (miRBase release 18.0) [12]. Here, we explore the miRNAs in our sequencing data to systematically identify both conserved and species-specific miRNAs in rose.

To identify conserved miRNAs in rose, we aligned the small RNA sequences against the known plant mature miRNAs registered in the miRBase (Release 17, April 2011), and their corresponding precursor sequences were checked to insure the miRNAs have their expected secondary structures. The aligned sRNAs of 18–24 nt long and having abundance of no less than 5 RPM (reads per million) in at least one library were regarded as the miRNA candidates and used for further analysis. A total of 33 known miRNA families were identified and listed in Table 1. Among them, 27 families were known and well-conserved, including *miR156/miR157*, *miR159*, *miR160*, *miR162*, *miR164*, *miR166*, *miR167*, *miR168*, *miR169*, *miR171*, *miR172*, *miR319*, *miR390*, *miR393*, *miR394*, *miR395*, *miR396*, *miR397*, *miR398*, *miR399*, *miR403*, *miR408*, *miR477*, *miR482*, *miR535*, *miR827*, and *miR858*. Six families were known but not well-conserved, including *miR2109*, *miR2478*, *miR4414*, *miR5072*, *miR5077*, and *miR5139*. In addition, the corresponding miRNA* sequences were detected in 26 families, further supporting the existence of these families in rose (Table 1). Unexpectedly, rose has several less-conserved miRNAs which were found previously only in monocots, such as *miR5072* and *miR5077*. In addition, *miR2109* and *miR4414*, which were specific in legume plants in previous reports, were also discovered in rose. The 5′ end of 48.5% (16 of 33) and 18.2% known miRNA families appeared predominantly as uridine (U) and adenosine (A), which were specifically generated by different Dicer proteins and recognized by Argonaute 1 and 2 proteins, respectively [33], [34].

**Table 1:** Known microRNAs identified from rose petals. 5′ end indicated the base frequency at the miRNA 5′ end.

Family	Size range	5′ end				Star(*)						
		A	U	C	G		*ath*	*ctr*	*osa*	*ptc*	*vvi*	*zma*
**Well-conserved**												
*miR156/miR157*	18–24	0.00	0.96	0.00	0.03	Yes	Yes	No	Yes	Yes	Yes	Yes
*miR159*	18–26	0.01	0.86	0.13	0.01	Yes	Yes	No	Yes	Yes	Yes	Yes
*miR160*	19–24	0.00	0.22	0.01	0.76	Yes	Yes	No	No	Yes	Yes	Yes
*miR162*	18–23	0.01	0.90	0.00	0.09	Yes	Yes	No	No	No	No	No
*miR164*	18–23	0.00	0.99	0.01	0.00	Yes	Yes	No	Yes	Yes	No	Yes
*miR166*	18–24	0.00	0.97	0.00	0.03	Yes	Yes	Yes	Yes	Yes	Yes	Yes
*miR167*	18–24	0.01	0.99	0.00	0.00	Yes	Yes	No	Yes	Yes	Yes	Yes
*miR168*	18–24	0.00	0.94	0.06	0.00	Yes	Yes	No	Yes	No	No	No
*miR169*	18–25	0.08	0.88	0.00	0.04	Yes	Yes	No	Yes	Yes	Yes	Yes
*miR171*	19–24	0.03	0.96	0.00	0.00	Yes	Yes	Yes	Yes	Yes	Yes	Yes
*miR172*	19–23	0.95	0.03	0.00	0.02	Yes	Yes	No	Yes	Yes	Yes	Yes
*miR319*	20–22	0.08	0.92	0.00	0.00	No	No	No	No	Yes	Yes	No
*miR390*	19–23	0.97	0.00	0.02	0.00	Yes	Yes	No	No	No	No	No
*miR393*	19–22	0.93	0.07	0.00	0.00	Yes	Yes	No	Yes	No	No	No
*miR394*	20–21	0.32	0.66	0.02	0.01	Yes	Yes	No	No	Yes	Yes	Yes
*miR395*	18–24	0.01	0.10	0.78	0.11	Yes	Yes	No	Yes	Yes	Yes	Yes
*miR396*	18–25	0.00	0.35	0.06	0.59	Yes	Yes	No	Yes	Yes	Yes	Yes
*miR397*	19–24	0.30	0.59	0.11	0.00	Yes	Yes	No	Yes	Yes	No	Yes
*miR398*	18–23	0.01	0.24	0.74	0.01	Yes	Yes	No	Yes	No	No	No
*miR399*	18–24	0.03	0.86	0.01	0.10	Yes	Yes	No	Yes	Yes	Yes	Yes
*miR403*	19–22	0.05	0.95	0.00	0.00	Yes	Yes	No	No	No	No	No
*miR408*	18–24	0.91	0.01	0.09	0.00	Yes	Yes	No	No	No	No	Yes
*miR477*	19–24	0.82	0.09	0.08	0.01	Yes	No	No	No	Yes	Yes	No
*miR482*	18–24	0.00	0.97	0.03	0.00	Yes	No	No	No	Yes	Yes	No
*miR535*	19–24	0.00	1.00	0.00	0.00	No	No	No	No	No	No	No
*miR827*	21–24	0.00	1.00	0.00	0.00	Yes	Yes	No	No	No	No	No
*miR858*	18–22	0.00	0.04	0.95	0.00	Yes	Yes	No	No	No	No	No
**Less-conserved**												
*miR2109*	21	0.00	1.00	0.00	0.00	No	No	No	No	No	No	No
*miR2478*	18–20	0.60	0.20	0.20	0.06	No	No	No	No	No	No	No
*miR4414*	19–22	0.99	0.00	0.01	0.00	No	No	No	No	No	No	No
*miR5072*	18–24	0.07	0.38	0.27	0.28	No	No	No	Yes	No	No	No
*miR5077*	18–22	0.13	0.61	0.00	0.26	No	No	No	Yes	No	No	No
*miR5139*	18–23	0.33	0.23	0.21	0.24	No	No	No	No	No	No	No

Ath, Arabidopsis thaliana; ctr, Citrus trifoliate; osa, Oryza sativa; ptc, Populus trichocarpa; vvi, Vitis vinifera; zma, Zea mays.

**Figure 2:**
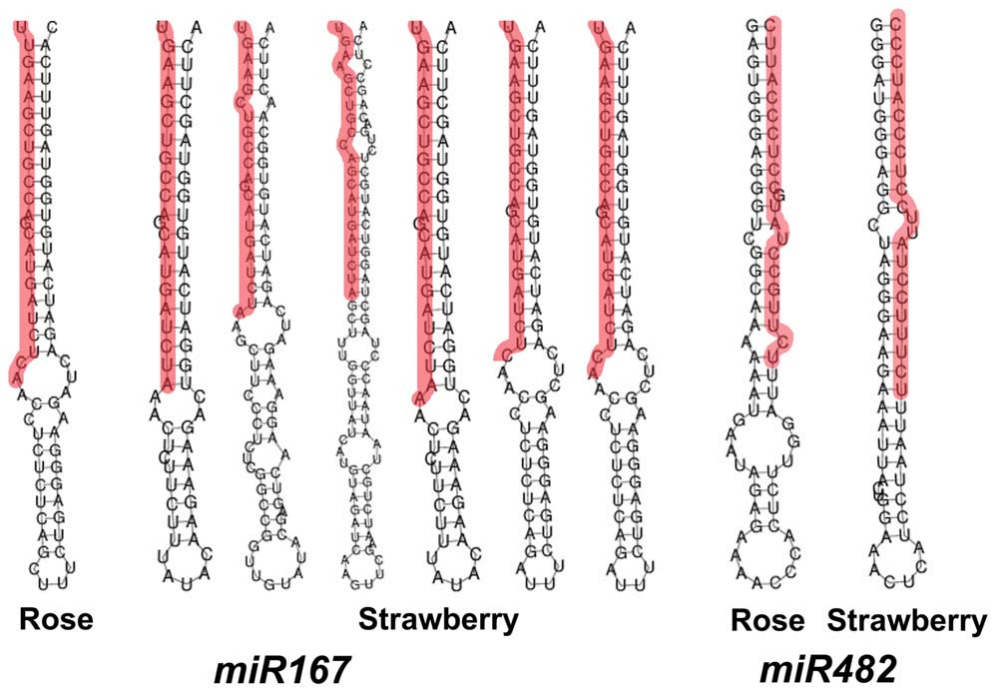
Predicted precursor structures of *miR167* and *miR482* in rose. The stem-loop structures were predicted by Vienna RNA software. miRNA sequences were highlighted in red.

MiRNA precursor prediction is an important step to identify the authentic miRNAs. Since currently the genomic information of rose is not available, genome sequence of a closely related plant, strawberry (*Fragaria vesca*) [35], which belongs to the subfamily *Rosoideae*, was used as the reference to predict rose miRNA precursors. As shown in Table 2, 92 precursors of 21 known miRNA families were predicted. We also predicted precursors of known miRNAs using the rose floral transcriptome sequences we generated as the reference (http://bioinfo.bti.cornell.edu/rose). However, precursors of only two miRNAs, *miR167* and *miR482*, were identified (Table 2 and Table S2). The failure of precursor prediction using the rose transcriptome database was mainly due to the fact that the database was constructed from poly (A) mRNAs, while miRNA precursors lack poly (A). The stem-loop structures of miRNA precursors of *miR167* and *miR482* predicted from rose and strawberry were almost identical (Figure 2), suggesting that they were highly conserved.

**Table 2:** Prediction of known miRNA precusors.

ID	miRNA	family	Precusor		
			ID	chr	folding energy
S066	UGACAGAAGAGAGUGAGCAC	miR156	H000126	LG3	−46.4
S066	UGACAGAAGAGAGUGAGCAC	miR156	H000127	LG3	−50.3
S066	UGACAGAAGAGAGUGAGCAC	miR156	H000128	LG2	−44.2
S066	UGACAGAAGAGAGUGAGCAC	miR156	H000129	LG3	−49.2
S066	UGACAGAAGAGAGUGAGCAC	miR156	H000130	LG2	−46
S067	UGACAGAAGAGAGUGAGCACA	miR156	H000131	LG3	−50.3
S067	UGACAGAAGAGAGUGAGCACA	miR156	H000132	LG2	−44.2
S067	UGACAGAAGAGAGUGAGCACA	miR156	H000133	LG3	−46.4
S067	UGACAGAAGAGAGUGAGCACA	miR156	H000134	LG3	−49.2
S067	UGACAGAAGAGAGUGAGCACA	miR156	H000135	LG2	−46
S068	UGACAGAAGAGAGUGAGCU	miR156	H000136	LG1	−45.6
S069	UGACAGAAGAGAGUGAGCUC	miR156	H000137	LG1	−45.6
S070	UGACAGAAGAGAGUGAGCUCA	miR156	H000138	LG1	−45.6
S071	UGACAGAAGAUAGAGAGCAC	miR156	H000139	LG3	−46.6
S071	UGACAGAAGAUAGAGAGCAC	miR156	H000140	LG3	−42.8
S071	UGACAGAAGAUAGAGAGCAC	miR156	H000141	LG5	−46.1
S090	UUGACAGAAGAGAGUGAGCAC	miR156	H000165	LG3	−47.3
S090	UUGACAGAAGAGAGUGAGCAC	miR156	H000166	LG3	−51.2
S090	UUGACAGAAGAGAGUGAGCAC	miR156	H000167	LG3	−50.1
S090	UUGACAGAAGAGAGUGAGCAC	miR156	H000168	LG2	−46.6
S093	UUGACAGAAGAUAGAGAGCACA	miR156	H000173	LG3	−47.4
S093	UUGACAGAAGAUAGAGAGCACA	miR156	H000174	LG5	−46.8
S091	UUGACAGAAGAUAGAGAGCA	miR156/miR157	H000169	LG3	−47.4
S091	UUGACAGAAGAUAGAGAGCA	miR156/miR157	H000170	LG5	−46.8
S092	UUGACAGAAGAUAGAGAGCAC	miR156/miR157	H000171	LG3	−47.4
S092	UUGACAGAAGAUAGAGAGCAC	miR156/miR157	H000172	LG5	−46.8
S099	UUUGGAUUGAAGGGAGCUCU	miR159	H000181	LG5	−74.5
S100	UUUGGAUUGAAGGGAGCUCUA	miR159	H000182	LG5	−75.5
S079	UGCCUGGCUCCCUGUAUGCCA	miR160	H000153	LG3	−46.2
S053	UCGAUAAACCUCUGCAUCCAG	miR162	H000101	LG5	−35.9
S080	UGGAGAAGCAGGGCACGUGCA	miR164	H000154	LG2	−40.8
S035	CGGACCAGGCUUCAUUCCCC	miR166	H000064	LG7	−40.36
S035	CGGACCAGGCUUCAUUCCCC	miR166	H000065	LG4	−42.6
S035	CGGACCAGGCUUCAUUCCCC	miR166	H000066	LG4	−43.2
S035	CGGACCAGGCUUCAUUCCCC	miR166	H000067	LG2	−50.7
S046	GGACCAGGCUUCAUUCCCC	miR166	H000084	LG2	−50.7
S046	GGACCAGGCUUCAUUCCCC	miR166	H000085	LG7	−40.36
S046	GGACCAGGCUUCAUUCCCC	miR166	H000086	LG4	−42.6
S046	GGACCAGGCUUCAUUCCCC	miR166	H000087	LG4	−43.2
S057	UCGGACCAGGCUUCAUUCC	miR166	H000105	LG7	−33.76
S057	UCGGACCAGGCUUCAUUCC	miR166	H000106	LG4	−36
S057	UCGGACCAGGCUUCAUUCC	miR166	H000107	LG4	−36.6
S057	UCGGACCAGGCUUCAUUCC	miR166	H000108	LG2	−44.1
S058	UCGGACCAGGCUUCAUUCCC	miR166	H000109	LG7	−37.06
S058	UCGGACCAGGCUUCAUUCCC	miR166	H000110	LG4	−39.3
S058	UCGGACCAGGCUUCAUUCCC	miR166	H000111	LG4	−39.9
S058	UCGGACCAGGCUUCAUUCCC	miR166	H000112	LG2	−47.4
S059	UCGGACCAGGCUUCAUUCCCC	miR166	H000113	LG2	−50.7
S059	UCGGACCAGGCUUCAUUCCCC	miR166	H000114	LG7	−40.36
S059	UCGGACCAGGCUUCAUUCCCC	miR166	H000115	LG4	−42.6
S059	UCGGACCAGGCUUCAUUCCCC	miR166	H000116	LG4	−43.2
S060	UCGGACCAGGCUUCAUUCCCCU	miR166	H000117	LG7	−41.96
S060	UCGGACCAGGCUUCAUUCCCCU	miR166	H000118	LG2	−52.3
S088	UUCGGACCAGGCUUCAUUCCC	miR166	H000163	LG2	−47.4
S062	UGAAGCUGCCAGCAUGAUCUA	miR167	H000120	LG1	−32.2
S062	UGAAGCUGCCAGCAUGAUCUA	miR167	H000121	LG2	−33.6
S062	UGAAGCUGCCAGCAUGAUCUA	miR167	H000122	LG2	−43.4
S063	UGAAGCUGCCAGCAUGAUCUAA	miR167	H000123	LG1	−32.2
S064	UGAAGCUGCCAGCAUGAUCUC	miR167	H000124	LG4	−38.2
S065	UGAAGCUGCCAGCAUGAUCUCA	miR167	H000125	LG4	−38.2
R13	UGAAGCUGCCAGCAUGAUCUC	miR167	H000031	RU29562	−37.2
R14	UGAAGCUGCCAGCAUGAUCUCA	miR167	H000032	RU29562	−37.2
S034	CGCUUGGUGCAGGUCGGGAA	miR168	H000063	LG5	−43.2
S054	UCGCUUGGUGCAGGUCGGGA	miR168	H000102	LG5	−44.7
S055	UCGCUUGGUGCAGGUCGGGAA	miR168	H000103	LG5	−44.7
S074	UGAGCCAAGGAUGACUUGCCU	miR169	H000144	LG4	−36.8
S076	UGAUUGAGCCGUGCCAAUAUC	miR171	H000146	LG5	−37.3
S076	UGAUUGAGCCGUGCCAAUAUC	miR171	H000147	LG3	−39.5
S076	UGAUUGAGCCGUGCCAAUAUC	miR171	H000148	LG5	−43.1
S076	UGAUUGAGCCGUGCCAAUAUC	miR171	H000149	LG2	−39.4
S076	UGAUUGAGCCGUGCCAAUAUC	miR171	H000150	LG6	−38.2
S095	UUGAGCCGUGCCAAUAUCACA	miR171	H000176	LG5	−45.5
S095	UUGAGCCGUGCCAAUAUCACA	miR171	H000177	LG3	−41.4
S039	GAAUCUUGAUGAUGCUGCAU	miR172	H000074	LG7	−45.5
S039	GAAUCUUGAUGAUGCUGCAU	miR172	H000075	LG3	−42.5
S039	GAAUCUUGAUGAUGCUGCAU	miR172	H000076	LG2	−35
S003	AAGCUCAGGAGGGAUAGCGCC	miR390	H000005	LG6	−38.6
S096	UUGGCAUUCUGUCCACCUCC	miR394	H000178	LG2	−38.5
S086	UUCCACAGCUUUCUUGAACUG	miR396	H000160	LG1	−50.3
S086	UUCCACAGCUUUCUUGAACUG	miR396	H000161	LG3	−36.7
S087	UUCCACAGCUUUCUUGAACUU	miR396	H000162	LG1	−41
S025	AUUGAGUGCAGCGUUGAUGAA	miR397	H000053	LG3	−49.5
S030	CAUUGAGUGCAGCGUUGAUGA	miR397	H000059	LG3	−49.9
S052	UCAUUGAGUGCAGCGUUGAUG	miR397	H000100	LG3	−49.9
S036	CGUGUUCUCAGGUCGCCCCUG	miR398	H000068	LG3	−73.7
S078	UGCCAAAGGAGAGUUGCCCUG	miR399	H000152	LG5	−43
S024	AUGCACUGCCUCUUCCCUGGC	miR408	H000052	LG1	−50.6
S077	UGCACUGCCUCUUCCCUGGCU	miR408	H000151	LG1	−50.6
S011	ACUCUCCCUCAAGGGCUUCUC	miR473	H000019	LG5	−54
S061	UCUUUCCUAUUCCUCCCAUCCC	miR482	H000119	LG5	−40.9
R12	UCUUGCCUAUGCCUCCCAUUCC	miR482	H000030	RU41075	−28.5
S084	UUAGAUGACCAUCAACAAACA	miR827	H000158	LG1	−31.8

The rose floral transcriptome database, and the genome sequence of strawberry (*F.vesca*) and transcriptome data of rose were used as reference, respectively. R, rose; S, strawberry. Detailed information is listed in Table S2.

### Identification of Novel miRNAs in Rose Petals

The genome sequences of strawberry and transcriptome sequences of rose (http://bioinfo.bti.cornell.edu/rose) were also used to predict the potential novel miRNAs in rose. According to the criteria for miRNA annotation [36], we used 5 RPM as cutoff to get rid of miRNAs with low expression level. In addition, for the cases of novel miRNAs without star sequences, we required the candidate miRNAs to be present in all four independent libraries. A total of 47 novel miRNA families were obtained and named as *rhy-miRC1* to *rhy-miRC47* (Table 3). Of the 47 novel miRNAs, *rhy-miRC1* was predicted from both rose and strawberry, while 8 and 38 miRNAs were predicted from rose and strawberry, respectively. The predicted hairpin structures of these novel miRNAs arranged from 61 to 242 nt in length and the folding energies were−18.4 to 106.6 ΔG (Table 3; Table S3). In addition, 16 out of the 47 novel miRNAs were predicted from more than one locus, suggesting that these miRNAs might be composed of multiple members (Table S3). Furthermore, the corresponding miRNA* sequences were identified for 27 novel miRNAs families, further supporting their existence as miRNAs (Table 3).

**Table 3:** Prediction of novel miRNA and their precusors.

Family	Length (nt)	ID	Sequence	Star (*)
*rhy-miRC1*	24	R1	AAGGGACUAGCAAAAGCUAAGUGU	Yes
	24	R5	AGGGACUAGCAAAAGCUAAGUGUG	
	24	S004	AAGGGACUAGCAAAAGCUAAGUGU	
	24	S019	AGGGACUAGCAAAAGCUAAGUGUG	
*rhy-miRC2*	21	R3	AGGGAAAAGCAUAGGAAUGAG	Yes
	22	R4	AGGGAAAAGCAUAGGAAUGAGU	Yes
*rhy-miRC3*	21	R16	UGGGAUGGGAAGAAUGGCACG	
	22	R17	UGGGAUGGGAAGAAUGGCACGA	
	23	R18	UGGGAUGGGAAGAAUGGCACGAA	
	22	R8	AUGGGAUGGGAAGAAUGGCACG	
*rhy-miRC4*	21	S007	AAUUUGGUGAUCGUUAAGGCA	
	23	S015	AGCCAAUUUGAUGAUCGUUAAGGC	Yes
	24	S016	AGCCAAUUUGGUGAUCGUUAAGGCA	Yes
	22	S027	CAAUUUGGUGAUCGUUAAGGCA	
	23	S042	GCCAAUUUGGUGAUCGUUAAGGCA	Yes
*rhy-miRC5*	24	S013	AGAUGAUCUAUACACUAGUACCUA	
	24	S014	AGAUGAUCUAUACAUUAGUACCUA	Yes
*rhy-miRC6*	20	S017	AGGCAGUCACCUUGGCUAAC	Yes
	21	S018	AGGCAGUCACCUUGGCUAACU	Yes
	19	S048	GGCAGUCACCUUGGCUAAC	Yes
*rhy-miRC7*	20	S038	CUCAAGAAAGCUGUGGGACA	Yes
	21	S044	GCUCAAGAAAGCUGUGGGACA	Yes
*rhy-miRC8*	21	S040	GAAUGUCGUCUGGUUCGAAAU	Yes
	22	S041	GAAUGUCGUCUGGUUCGAAAUC	Yes
*rhy-miRC9*	20	S072	UGACGAUGAGAGAGAGCACG	
	21	S073	UGACGAUGAGAGAGAGCACGC	
	21	S094	UUGACGAUGAGAGAGAGCACG	
*rhy-miRC10*	19	S082	UGUAUGUUCGUCUCCAACU	
	21	S083	UGUAUGUUCGUCUCCAACUCU	
*rhy-miRC11*	24	S026	AUUUUCAGCCAAAUUGAUGAUCGU	
	21	S085	UUCAGCCAAAUUGAUGAUCGU	
	21	S097	UUUCAGCCAAAUUGAUGAUCG	Yes
*rhy-miRC12*	21	S102	UUUUCUGAUUGAGCCGUGCCA	Yes
	21	S103	UUUUUCUGAUUGAGCCGUGCC	Yes
*rhy-miRC13*	21	R2	ACAUGGAACACUACGACAUGG	Yes
*rhy-miRC14*	21	R6	AGUGGGAGGGUCGGCAAAAAA	Yes
*rhy-miRC15*	24	R7	AUGAUUGUGGAUAGAUUAAGCAUG	
*rhy-miRC16*	21	R10	GAGAUGGAGAUGGAGAGCUAG	
*rhy-miRC17*	21	R11	GCAUUCCUAUGCUUUUUCUCCA	Yes
*rhy-miRC18*	21	R15	UGGAUGCUUUGGAUGGAACGG	Yes
*rhy-miRC19*	21	S001	AAAUUGAUGAUCGUUAAGGUA	
*rhy-miRC20*	24	S002	AAGCCAAAUUGGUGAUCGUUAAGG	
*rhy-miRC21*	21	S005	AAUAAAGCUGUGGGAAGAUAC	Yes
*rhy-miRC22*	24	S006	AAUAUUACUAUUUUGAGGACUCAU	
*rhy-miRC23*	24	S008	ACAGGCGGUGGAACAAAUAUGAAU	
*rhy-miRC24*	21	S009	ACCUAGCUCUGAUACCAUGUG	Yes
*rhy-miRC25*	22	S010	ACUCUCCCUCAAGGGCUUCUAG	
*rhy-miRC26*	21	S012	AGAAUCUUGAUGAUGCUGCAU	Yes
*rhy-miRC27*	21	S020	AGUGGAGUUCUGGGAAAGAAG	
*rhy-miRC28*	24	S021	AGUUGGGACAAUAUCGGUACAAUG	
*rhy-miRC29*	24	S022	AGUUUUAAGGGACUGUGAGGGACA	
*rhy-miRC30*	21	S023	AUCAUGCUAUCCCUUUGGAUU	Yes
*rhy-miRC31*	21	S028	CAGGUCGGGAACUGCUUCGGU	
*rhy-miRC32*	21	S029	CAUCAACGCUGCACCCAAUUA	Yes
*rhy-miRC33*	21	S031	CCCGCCUUGCAUCAACUGAAU	Yes
*rhy-miRC34*	21	S032	CGAGCCGAACCAAUAUCACUC	
*rhy-miRC35*	21	S033	CGCUAUCCAUCCUGGGUUUCC	Yes
*rhy-miRC36*	21	S037	CUAGUCAUUGGUCAUAGCAUC	
*rhy-miRC37*	21	S043	GCGUACGAGGAGCCAAGCAUA	Yes
*rhy-miRC38*	21	S045	GCUCUCUAUGCUUCUGUCAUC	Yes
*rhy-miRC39*	24	S047	GGAGUGUGGAUUGUAAAAUGGGGA	
*rhy-miRC40*	21	S049	GUUCAAUAAAGCUGUGGGAAG	Yes
*rhy-miRC41*	22	S050	UAUGUCGCAGGAGAGAUGGUAC	
*rhy-miRC42*	22	S051	UCAAUAAAGCUGUGGGAAGAUA	Yes
*rhy-miRC43*	22	S056	UCGCUUGGUGCAGGUCGGGAAC	Yes
*rhy-miRC44*	21	S081	UGGGAUUUGGCGAAUUGUGGU	Yes
*rhy-miRC45*	22	S089	UUCGGACCAGGCUUCAUUCCCC	Yes
*rhy-miRC46*	21	S098	UUUGAAGUGGGAUUUGGCGAA	
*rhy-miRC47*	24	S101	UUUGGCUGAAAUUUUGCAGAGAUG	

The rose floral transcriptome database, and the genome sequence of strawberry (F.vesca) and transcriptome data of rose were used as reference, respectively. R, roses; S, strawberry.

**Figure 3:**
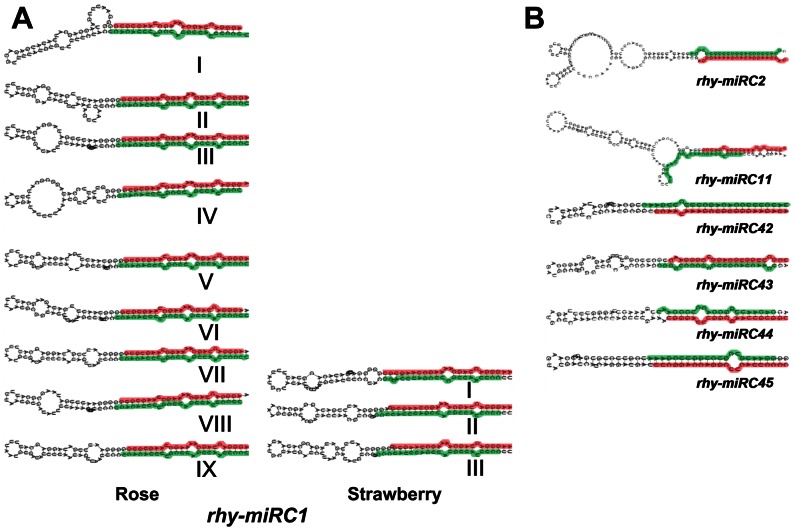
Predicted precursor structures of novel miRNAs in rose. Precursor stem-loop structures of novel miRNAs predicted based on rose transcriptome (A) or genome sequence of strawberry (*F. vesca*) (B) are displayed. The mature miRNA sequences are highlighted in red and miRNA* sequences in green.

We compared the stem-loop structures of *rhy-miRC1* predicted from rose and strawberry. As shown in Figure 3A, the precursor structures were much more similar between these two species, indicating that rose and strawberry possess the same miRNAs which have not been reported in other plant species until now. In addition, the stem-loop structures of *rhy-miRC2/11/42/43/44/45* were presented in Figure 3B.

### Abundance of Conserved and Novel MiRNAs in Rose Petals

Since high-throughput sequencing provides the opportunity for quantitative profiling of sRNA populations, the sequencing frequency has been used to estimate the miRNA abundance in different samples. Nearly half of the conserved miRNA families were represented with more than 50 RPM in at least one library, including *miR156*, *miR157*, *miR159*, *miR164*, *miR166*, *miR167*, *miR168*, *miR171*, *miR172*, *miR390*, *miR396*, *miR397*, *miR408*, *miR535*, and *miR827*. On the other hand, all six known but less-conserved families (*miR2109*, *miR2478*, *miR4414*, *miR5072*, *miR5077*, and *miR5139*) exhibited high abundance, namely more than 50 RPM in at least one library (Table S4). *MiR156*, *miR157*, *miR166* and *miR168* were the most highly expressed conserved miRNAs. The highest expression level of *miR159*, *miR164*, *miR166*, *miR167*, *miR397*, *miR408* and *miR827* were observed in petals of unopened buds (S0), indicating that they may play roles in earlier period of flower opening; whereas, *miR168*, *miR171* and *miR390* were enriched in petals of opened flowers (C24). In addition, *miR156*, *miR157*, *miR535* and *miR2109* were highly accumulated in petals of ethylene-treated flowers (E24). Of the 47 novel miRNAs, 12 appeared to be highly expressed in petals (more than 50 RPM in at least one library) (Table S4). *Rhy-miRC3*, *rhy-miRC9* and *rhy-miRC26* were the most highly expressed novel miRNAs. Moreover, *rhy-miRC3* and *rhy-miRC26* were highly expressed in petals of unopened buds (S0), while *rhy-miRC2*, *rhy-miRC9*, *rhy-miRC17*, *rhy-miRC34*, and *rhy-miRC41* were enriched in petals of ethylene-treated flowers (E24).

Generally, the miRNA* sequences are considered to be quickly degraded after their complementary miRNA sequences are selected from the miRNA/miRNA* duplex and loaded into the AGO protein [1], [2]. Therefore, the abundance of miRNA* is usually much lower than that of their corresponding miRNAs. However, we noticed that the abundance of miRNA* of two conserved miRNA families, *miR171* and *miR396*, was much higher (Figure S1), which was consistent with the finding in *Brassica* [37].

In summary, our analysis showed that both known and novel miRNAs exhibited highly diverse expression patterns during flower opening and in response to ethylene, indicating that they may play different roles in these biological processes.

### Prediction and Validation of MiRNA Targets in Rose Petals

To understand possible biological functions of the identified miRNAs in rose, we identified their putative targets using the rose floral transcriptome database as the reference transcript set (http://bioinfo.bti.cornell.edu/rose). Putative targets of 28 known miRNA families were predicted (Table 4 and 5; Table S5) and all the well-conserved miRNAs, such as *miR156*, *miR159*, *miR160*, *miR164*, *miR167*, *miR172*, *miR396*, *miR397*, and *miR482* shared conserved target genes with their homologous miRNAs in other plants (Table 4), indicating that these miRNAs might play a fundamental role in plant development. Interestingly, we also identified some novel targets of both conserved and less-conserved known miRNA families (Table 5; Table S5). These putative novel targets included several regulatory proteins, such as protein kinase (*miR156*), E3 ubiquitin protein (*miR159*), enhancer of mRNA-decapping protein (*miR169*), zinc finger protein (*miR172, miR394* and *miR5139*), and RING-H2 finger protein (*miR397*). Moreover, we also found a lot of structure and metabolism proteins, including DXPS3 (1-deoxy-D-xylulose-5-phosphate synthase 3) (*miR156*), cytokinin oxidase (*miR159*), pectin methylesterase (*miR166*), beta-galactosidase (*miR166*), hydrolase (*miR167*), pentatricopeptide (PPR) repeat-containing protein (*miR2478*), and expansin (*miR5139*) (Table S5).

**Table 4:** Predicted targets for conserved miRNAs in rose.

miRNA family	Targets ID	Targets Annotation
*miR156*	RU15050	SQUAMOSA PROMOTER BINDING PROTEIN-LIKE
	RU39321	
	RU35697	
*miR159*	RU13577	R2R3-myb transcription factor
*miR160*	RU26455	Auxin response factor
*miR164*	RU24899	NAC domain protein
	RU60879	
	RU02822	
*miR168*	RU01155	Argonaute protein
*miR172*	RU08179	Transcription factor AHAP2
	RU01226	AINTEGUMENTA protein
*miR319*	RU00790	TCP transcription factor
*miR397*	RU16913	Laccase-like protein
*miR482*	RU12701	NBS-LRR type resistance protein

**Table 5:** Predicted new targets for known miRNAs in rose.

miRNA family	Targets ID	Targets Annotation
*miR156*	RU23956	E3 ubiquitin protein ligase UPL1, putative
*miR156*	RU41939	Nucleic acid binding protein, putative
*miR156*	RU54762	Protein kinase
*miR156*	RU20461	Timing of CAB expression 1 protein
*miR156/miR157*	RU20216	Guanine nucleotide-exchange-like protein
*miR156/miR157*	RU06764	RING/U-box domain-containing protein
*miR159*	RU24195	Cytokinin dehydrogenase
*miR159*	RU48935	ATP-binding region, ATPase-like domain-containing protein
*miR159*	RU17672	Glycosyl transferase family 17 protein
*miR166*	RU06269	beta-galactosidase
*miR166*	RU10736	Kinesin-like protein
*miR166*	RU03738	Pectin methylesterase 1
*miR166*	RU14941	*Rosa rugosa* Rdr1 homologous region genomic sequence
*miR168*	RU60766	U-box domain-containing protein 43-like
*miR169*	RU34168	CCAAT-binding transcription factor subunit B
*miR169*	RU00770	*Rosa multiflora* breeding line 88/124–46 black spot resistance muRdr1 gene locus
*miR169*	RU09780	Nucleotide binding
*miR171*	RU44711	Ubiquitin-protein ligase
*miR172*	RU24707	Peptidyl-prolyl *cis-trans* isomerase
*miR172*	RU60452	Zinc finger, C2H2-type
*miR394*	RU05839	Dehydration-responsive protein-related
*miR394*	RU21813	E3 ubiquitin-protein ligase
*miR394*	RU54433	*Rosa rugosa* Rdr1 homologous region genomic sequence
*miR394*	RU17225	Zinc finger (C3HC4-type RING finger)
*miR397*	RU01636	Putative RING-H2 finger protein RHF2a [Arabidopsis thaliana]
*miR398*	RU47741	Receptor protein kinase-like protein
*miR399*	RU52206	*Rosa multiflora* breeding line 88/124–46 black spot resistance muRdr1 gene locus
*miR482*	RU12701	NBS-LRR type resistance protein
miR2109	RU60115	*Rosa rugosa* Rdr1 homologous region genomic sequence
*miR2109*	RU27536	TIR-NBS type disease resistance protein
*miR4414*	RU22051	Putative cyclic nucleotide-gated cation channel
*miR4414*	RU44477	SGS3 (SUPPRESSOR OF GENE SILENCING 3)
*miR473*	RU33494	Putative methyltransferase
*miR5072*	RU23976	Putative auxin influx carrier protein
*miR5077*	RU25746	ARF GTPase activator
*miR5139*	RU22365	Expansin
*miR5139*	RU05060	Zinc finger, C3HC4 type

Putative targets of 26 out of the 47 novel miRNAs in rose were also predicted (Table S6). The predicted targets included several types of regulatory protein, such as transcription factor (*rhy-miRC16/rhy-miRC26/rhy-miRC27*), cell cycle factor (*rhy-miRC16/rhy-miRC44*) and component of ubiquitin-dependent protein degradation system (*rhy-miRC46*). Meanwhile, the novel targets also included a lot of functional proteins, such as glycosyl hydrolase (*rhy-miRC2*), ARPC1b (actin-related 18 protein C1b) (*rhy-miRC7*), plastid developmental protein (*rhy-miRC9*), UDP-glucuronate decarboxylase (*rhy-miRC10*) proton pump interactor (*rhy-miRC14*), auxin conjugate hydrolase (*rhy-miRC14*), phosphoenolpyruvate synthase (*rhy-miRC16*), acyl-CoA-binding protein (*rhy-miRC18*), dormancy/auxin associated protein (*rhy-miRC22*), ATPase (*rhy-miRC41*), argonaute protein (*rhy-miRC43*), and actin binding protein (*rhy-miRC44*). Candidate targets of eight novel *miRNA* families, including *rhy-miRC1*, *rhy-miRC4*, *rhy-miRC5*, *rhy-miRC11*, *rhy-miRC19*, *rhy-miRC20*, *rhy-miRC24* and *rhy-miRC47*, failed to be annotated (Table S6). The lack of functional annotation of these putative targets indicated that they might be novel target genes which were specific in roses.

**Figure 4:**
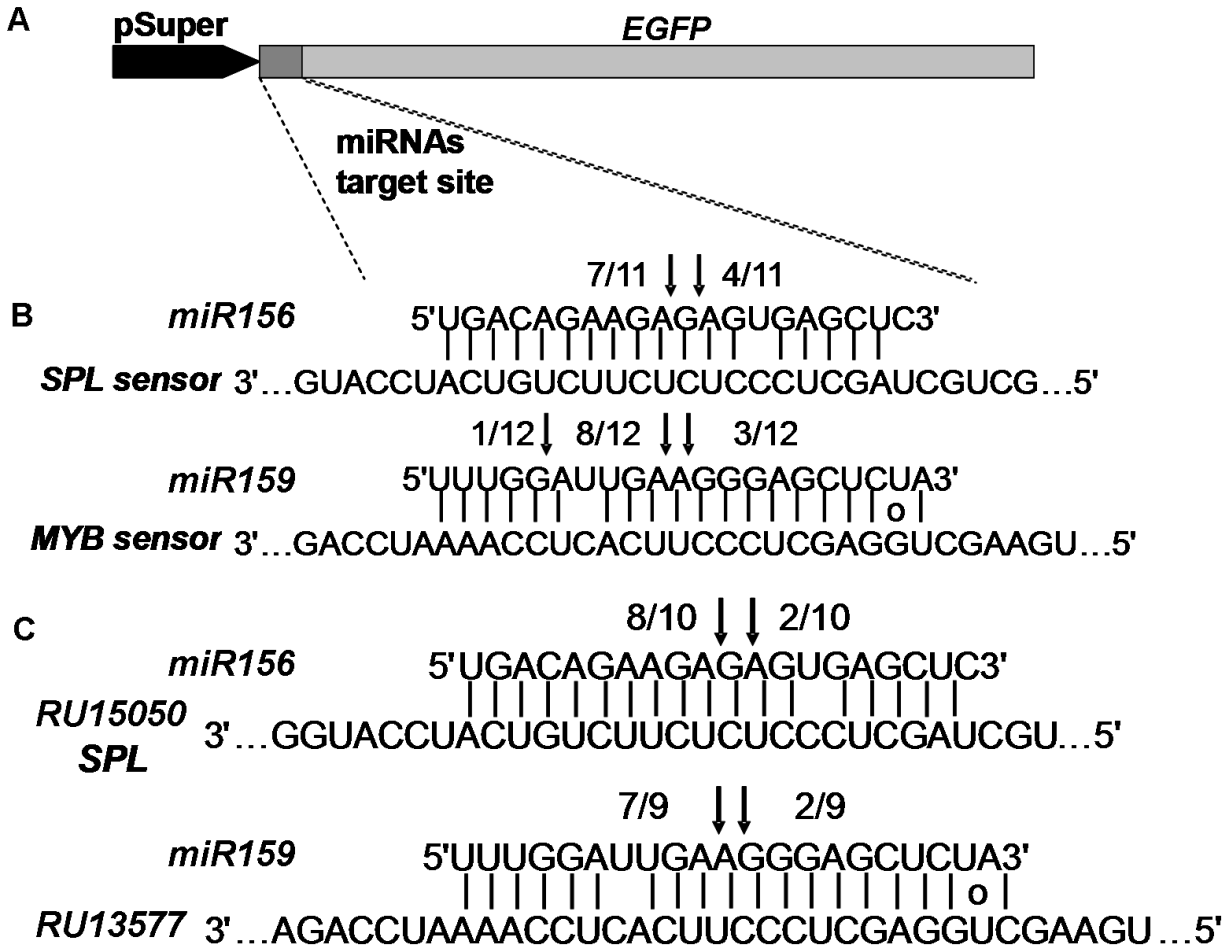
Validation of miRNA predicted targets. (A) Constructs of miRNA target sensors. (B, C) Cleavage sites identified by 5′RLM-RACE assay in tobacco (B) and in rose petals (C). Positions of the cleavage sites are indicated by arrows with the proportion of sequenced clones.

We then performed 5′-RNA ligase-mediated (RLM)-RACE analysis to validate the miRNA-guided cleavage of predicted target transcripts. As reported previously, squamosa-promoter-binding protein-like (SPL) family genes and R2R3 MYB transcription factors were predicted as targets of *miR156* and *miR159*, respectively (Table 4) [3]. The putative miRNA-target sites of RU15050 (SPL 7 gene) and RU13577 (MYB) were fused with the ORF sequence of EGFP in frame and driven by a pSuper promoter to construct the SPL-sensor and MYB-sensor, respectively (Figure 4A). The sensor constructs were transformed into *Agrobacterium* and then co-infiltrated with *35S:miR156a* (for SPL sensor) and *35S:miR159a* (for MYB sensor), respectively. As expected, *miR156*- and *miR159*-mediated cleavage sites were detected in SPL and MYB sensors, respectively (Figure 4B). Meanwhile, we performed the 5′-RACE using total RNA extracted from rose petals and confirmed the *miR156*- and *miR159*-mediated cleavage of SPL (RU15050) and MYB (RU13577) gene *in vivo*, respectively (Figure 4C).

### MiRNA Profiling in Rose Petals during Early Flower Opening and in Response to Ethylene

Since the high-throughout sequencing data of small RNAs can be used to evaluate the miRNA expression profiles, we investigated miRNA profiles in petals during the early period of flower opening and in response to ethylene in rose. Except for *miR160*, *miR390*, *miR482*, *miR858* and *miR827*, all known miRNAs exhibited substantial expression changes during early flower opening (stage 2/stage 0≤0.67 or≥1.50): abundance of 10 miRNAs decreased, while 18 increased (Table 6). Of the 28 changed miRNAs, the most pronounced expression decrease (≤10-fold) was found in *miR4414* and *miR159*, while the increase≥10-fold) was found in *miR5139*, *miR394*, *miR396* and *miR473*. Among the 47 novel miRNAs, expression of 17 and 22 miNRAs substantially decreased (stage 2/stage 0≤0.67) and increased (stage 2/stage 0≥1.50), respectively, during early flower opening. The novel miRNAs whose abundance decreased more than 10-fold included *rhy-miRC45*, *rhy-miRC32* and *rhy-miRC37*, and the increased ones (≥10-fold) included *rhy-miRC21*, *rhy-miRC42*, *rhy-miRC40*, *rhy-miRC13* and *rhy-miRC30* (Table 6).

**Table 6:** Digital expression profiles of known and novel miRNAs in rose petals during earlier opening period and in response to ethylene.

miRNA ID	Stage2/Stage 0	+C_2_H_4_/−C_2_H_4_	miRNA ID	Stage 2/Stage 0	+C_2_H_4_/−C_2_H_4_
***miR156***	1.80	1.81	***rhy-miRC1***	0.48	0.53
***miR157***	2.53	1.58	***rhy-miRC2***	3.23	1.74
*miR159*	0.07	1.03	*rhy-miRC3*	0.35	1.34
***miR160***	0.90	0.35	***Rhy-miRC4***	2.94	0.67
***miR162***	0.45	0.38	***rhy-miRC5***	1.53	1.75
***miR164***	0.25	0.47	***rhy-miRC6***	3.98	0.48
***miR165***	0.44	0.34	***rhy-miRC7***	1.02	0.29
***miR166***	0.28	0.39	***rhy-miRC8***	0.81	0.52
***miR167***	0.25	0.51	*Rhy-miRC9*	1.81	1.11
***miR168***	1.64	0.66	*rhy-miRC10*	1.88	0.88
***miR169***	1.59	0.53	***rhy-miRC11***	0.50	0.25
***miR171***	1.63	0.40	***rhy-miRC12***	0.13	2.72
***miR172***	6.30	0.61	*rhy-miRC13*	5.84	0.98
*miR319*	0.00	/	*rhy-miRC14*	0.75	0.90
*miR390*	1.08	0.83	***rhy-miRC15***	0.23	2.52
***miR394***	10.77	2.52	*rhy-miRC16*	1.37	0.77
*miR395*	5.65	1.05	*rhy-miRC17*	2.20	1.15
***miR396***	10.36	0.37	*rhy-miRC18*	0.43	1.37
***miR397***	0.16	0.33	*rhy-miRC19*	0.30	0.73
***miR398***	2.35	0.56	*rhy-miRC20*	0.35	1.39
***miR399***	3.25	2.19	*rhy-miRC21*	43.92	1.08
***miR408***	0.19	0.37	*rhy-miRC22*	1.14	1.06
***miR473***	10.23	2.31	***rhy-miRC23***	3.57	2.22
***miR482***	1.15	0.30	***rhy-miRC24***	3.83	0.51
***miR535***	3.41	3.38	***rhy-miRC25***	7.17	0.42
*miR827*	0.79	0.78	*rhy-miRC26*	4.42	0.81
***miR858***	0.78	0.38	***rhy-miRC27***	0.40	2.18
***miR2109***	4.47	5.71	*rhy-miRC28*	0.30	0.88
***miR2478***	5.91	0.27	***rhy-miRC29***	0.54	0.43
***miR4414***	0.00	0.12	*rhy-miRC30*	14.90	1.17
***miR5072***	7.75	0.33	***rhy-miRC31***	2.26	0.49
***miR5077***	3.30	0.33	*rhy-miRC32*	0.06	1.12
***miR5139***	22.93	0.49	*rhy-miRC33*	4.47	0.75
			***rhy-miRC34***	1.12	1.55
			*rhy-miRC35*	4.01	0.73
			***rhy-miRC36***	1.17	0.35
			***rhy-miRC37***	0.08	4.33
			*rhy-miRC38*	4.03	1.19
			*rhy-miRC39*	5.91	0.94
			***rhy-miRC40***	22.17	0.56
			***rhy-miRC41***	1.17	45.72
			*rhy-miRC42*	30.67	0.86
			*rhy-miRC43*	1.50	0.93
			*rhy-miRC44*	0.42	0.92
			***rhy-miRC45***	0.01	0.34
			*rhy-miRC46*	0.50	1.40
			*rhy-miRC47*	0.64	0.97

For ethylene treatment, flowers (stage 2) were treated with 10 ppm ethylene in a sealed airtight chamber for 24 h, and flowers exposed to air were used as the control. The miRNAs in bold indicate miRNAs showing substantial expression changes in response to ethylene treatment (+C_2_H_4_/−C_2_H_4_≤0.67 or≥1.50).

To screen possible ethylene-sensitive miRNAs, we compared the expression level of miRNAs between flowers (stage 2) treated with or without 10 ppm ethylene for 24 h. We found that 28 out of 33 known miRNAs (84.8% of known miRNAs) showed substantial expression changes in response to ethylene treatment (**+**C2H4/**−**C2H4≤0.67 or≥1.50), while expression of 22 novel miRNAs (46.8% of novel miRNAs) was substantially changed after ethylene treatment. Interestingly, a novel miRNA, *rhy-miRC41*, exhibited a∼46-fold increase in its expression in ethylene-treated flower petals when compared to the control (Table 6).

**Figure 5:**
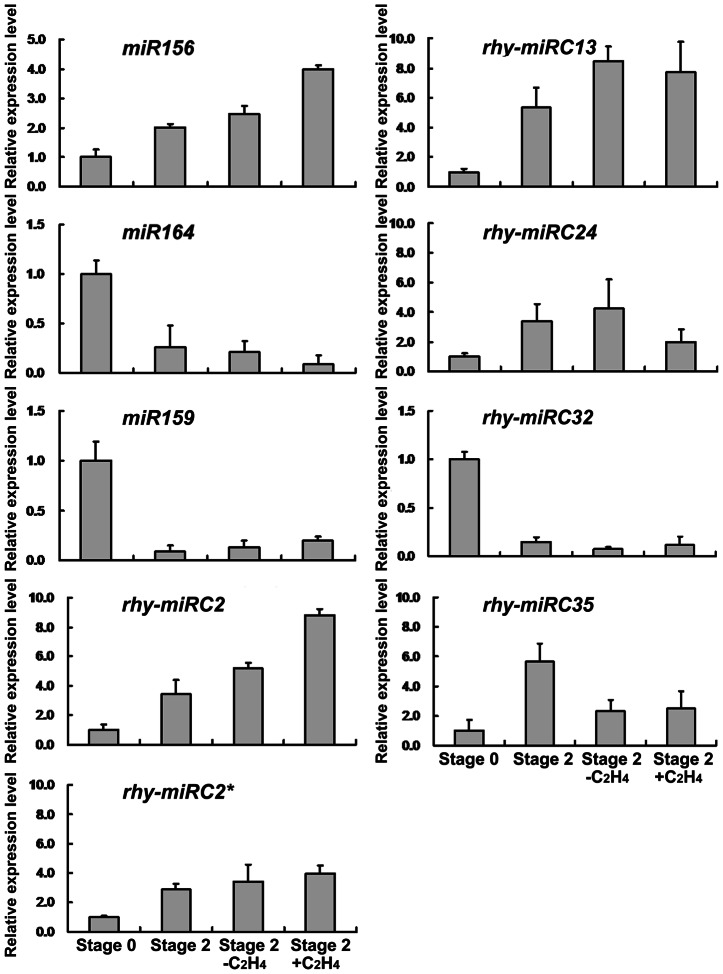
qRT-PCR of selected known and novel miRNAs differentially expressed in petals during earlier flower opening or in response to ethylene. 5S rRNA was used as the internal control. Error bars indicate the SD of three biological replicates.

We also performed quantitative RT-PCR to examine the expression of several known and novel miRNAs. The expression patterns obtained from qRT-PCR supported the sequencing data. More importantly, qRT-PCR results confirmed that expression of *miR156*, *rhy-miRC2*, *rhy-miRC13*, *rhy-miRC24*, and *rhy-miRC35* was substantially increased and expression of *miR159*, *miR164* and *rhy-miRC32* was sharply reduced in petals during the early flower opening. After ethylene treatment, abundance of *miR156* and *rhy-miRC2* was substantially increased, while *miR164* and *rhy-miRC24* decreased (Figure 5). In addition, we also detected the expression of rhy-miRC2*, further confirming the authenticity of the predicted novel miRNAs.

### Putative mRNA/miRNA Modules Involved in Ethylene-Regulated Flower Opening of Rose

**Figure 6:**
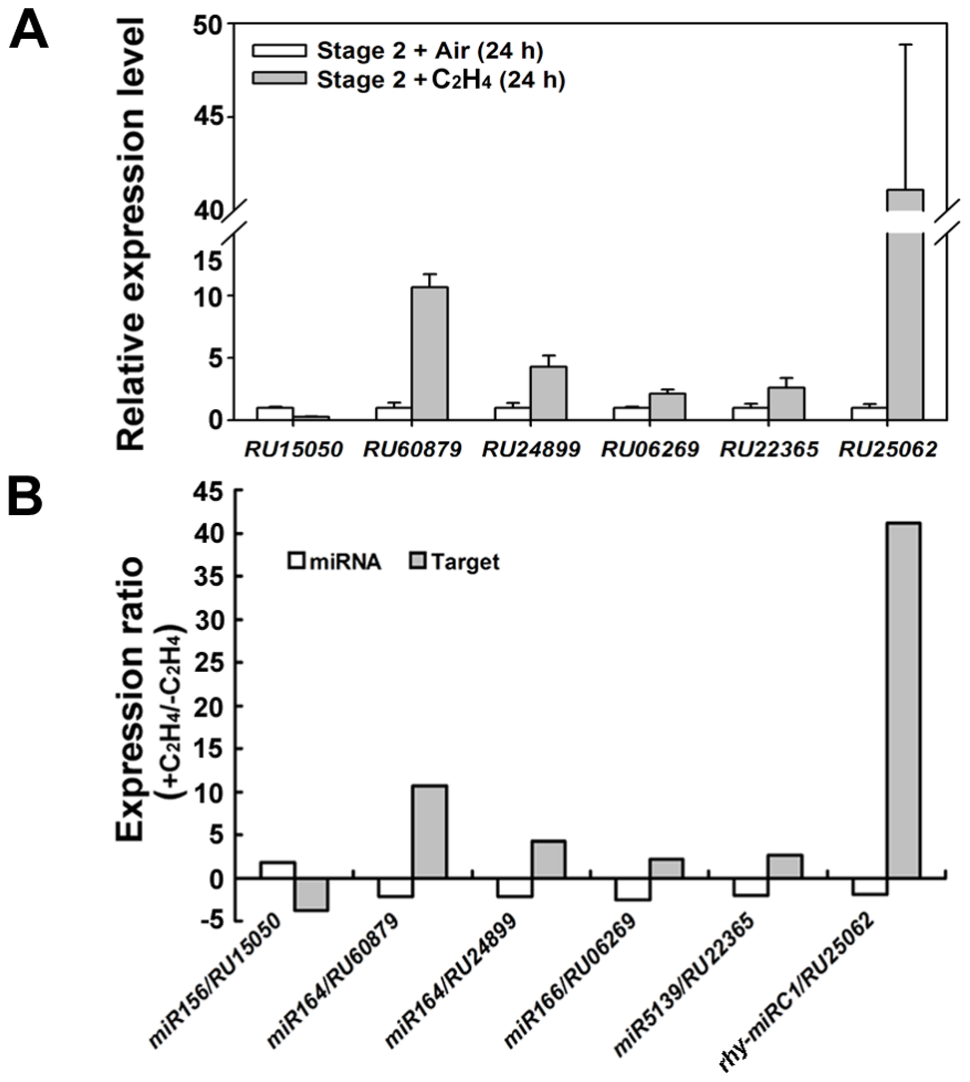
qRT-PCR of predicted miRNA targets (A) and expression ratio of miRNA/target modules in rose petals in response to ethylene (B). *RhACT5* was used as the internal control. Error bars indicate the SD of three biological replicates.

Recent reports showed that translation repression is an important and popular way for miRNAs to regulate their targets in plants, and it is likely that miRNAs repress their targets through both transcript cleavage and translation repression [38], [39]. However, integrative analysis of expression of miRNAs and their targets can still be helpful to identify miRNA/mRNA modules which might be involved in regulating specific biological processes. Here, we analyzed expression profiles of miRNAs and their predicted targets in rose petals treated with or without ethylene and identified 75 putative miRNA/mRNA modules, which included 21 known and 16 novel miRNAs (Table S7). Of these 75 miRNA/mRNA modules, expression of 5 miRNAs, including *miR156*, *miR164*, *miR166*, *miR5139* and *rhy-miRC1*, were inversely correlated to that of their 7 corresponding targets in response to ethylene treatment (Table 7). We also tested the expression changes of predicted targets in response to ethylene in rose petals by using quantitative RT-PCR. As shown in Figure 6, qRT-PCR further confirmed that the expression of miRNAs and their predicted targets was inversely correlated.

**Table 7:** Integrated analysis of expression profiles of miRNAs and responsed targets in rose petals.

miRNA ID	Target ID	Accession	Best Hit(nr_hit)	E-value	miRNA expression +C2H4/−C2H4	Target expression +C2H4/−C2H4		Score
						Fold change	FDR	
*miR156*	RU15050	NP_175723	SPL4 (SQUAMOSA PROMOTER BINDING PROTEIN-LIKE 4); DNA binding/transcription factor [Arabidopsis thaliana]	8.00E-10	1.81	0.17	2.06E-03	3.0
*miR164*	RU02822	CAO15010	ANAC100	2.00E-58	0.47	15.83	3.41E-03	2.5
	RU24899	ACI13682	NAC domain protein [Malus x domestica]	3.00E-60	0.47	2.51	2.86E-01	2.5
	RU60879	ACI13682	NAC domain protein [Malus x domestica]	1.00E-43	0.47	13.32	1.24E-08	2.5
*miR166*	RU06269	CAC44500	beta-galactosidase [Fragaria x ananassa]	2.00E-245	0.39	1.83	6.37E-02	3.0
*miR5139*	RU22365	AAD44345	expansin [Fragaria x ananassa]	9.00E-17	0.49	3.07	7.08E-05	3.0
*rhy-miRC1*	RU25062		No hit		0.53	50.88	1.22E-03	0.5

For ethylene treatment, flowers (stage 2) were treated with 10 ppm ethylene in a sealed airtight chamber for 24 h, and flowers exposed to air were used as the control.

## Discussion

### Known and Novel miRNAs in Rose Petals

*Rosaceae* plant is one of the six most economically important crop plant families, and includes many important fruits and ornamental plants, such as apple, pear, almond, peach, apricot, plum, cherry, strawberry, and rose [40]. As one of the most important ornamental crops, rose accounts for more than 30% of cut flower trade in Europe and China (Data from MOA, 2011) [18]. Aside from the economic significance, rose has also served as a model system to investigate some important biological processes, such as fragrance formation and flower opening. Therefore, understanding the molecular mechanism in shaping flower ornamental quality in rose will provide great value for rose production and breeding.

miRNAs have fundamental functions in regulating almost all aspects of plant development. In the present study, we performed small RNA sequencing and determined the expression profiles of miRNAs in rose petals in response to ethylene. We identified 47 novel and 33 known miRNA families, as well as their corresponding star sequences. Due to the lack of rose genome sequence, the precursors were predicted using both strawberry genome and our rose transcriptome sequences. Although we obtained precursors of *miR166* and *miR482* from the rose transcriptome sequences, their precursors were much more similar to those predicted using the strawberry genome.

A recent study reported 25 novel and 242 known miRNA sequences identified from flowers of three modern rose cultivars and *Rosa rugosa* [28]. Based on our analysis, we found that the reported 242 known miRNAs could be categorized into 37 miRNA families (Table S9), and 29 families were also identified by us. Five families (*miR828*, *miR845*, *miR2111*, *miR2275* and *miR2911*) were not listed by us due to their low expression levels (<5 RPM), but found in our sequencing data. Four known miRNA families (*miR2478*, *miR5072*, *miR5077* and *miR5139*) were identified from our sRNA dataset, but were not listed in Kim et al [28].

For novel miRNAs, 17 predicted novel miRNAs were reported by Kim et al [28], while 47 novel miRNAs were identified in our work. Although both Kim et al and us used strawberry (*Fragaria vesca*) genome to predict the novel miRNAs, only five (*rhy-miRC4/ng9*, *rhy-miRC10/ng4*, *rhy-miRC24/ng8*, *rhy-miRC27/ng11* and *rhy-miRC36/ng6*) were presented in both works. In addition, seven other novel miRNAs (*ng1*, *ng5*, *ng12*, *ng13*, *ng14*, *ng15* and *ng16*) in Kim et al [28] were also found in our sequencing data, but were not regarded as authentic miRNAs due to their low abundance (<5 RPM) in our samples (Table S9). Therefore, a total of 12 and 42 miRNAs were specific in Kim's and our dataset, respectively. Considering that the sampling strategies were different, the difference in novel miRNAs could also be attributed to the cultivar-, organ-, development- and/or condition-specific expression pattern of miRNAs. The five identical rose-specific miRNAs are likely to play conserved roles in different rose cultivars (Table S9).

Using a rose floral transcriptome database, we identified putative targets of rose miRNAs. The well known target genes of most conserved miRNAs, such as *miR156*, *miR164*, and *miR172*, have also been identified in rose. However, 32.5% (39/120) predicted targets of known miRNAs, and 48.4% (45/93) of novel miRNAs, were not homologous to any proteins in the GenBank nr database, indicating that they might be novel genes which were specific in rose. Interestingly, a novel miRNA, *rhy-miRC27*, was predicted to target an acyltransferase-like protein (RU23954) (Table S7), which was also identified as the target of *ng11*, a miRNA identical to *rhy-miRC27*, in a previous report [28]. These results suggested that *rhy-miRC27* (*ng11*)/acyltransferase module might be conserved in *Rosa* sp.

### MiRNA Profiling during Early Flower Opening and in Response to Ethylene

In the present study we investigated expression profiles of miRNAs during early flower opening and in response to ethylene in rose petals. We found that much larger portion of known miRNAs than novel ones appeared to be differentially expressed during early opening stages or regulated by ethylene treatment. This is consistent with recent findings that ethylene biosynthesis and signaling pathway emerged in earlier period of land plant evolution [41], [42], thus the non-conserved novel miRNAs are supposed to occur later evolutionarily [2], [4].

Recently, a report showed that ACC (1-aminocyclopropane-1-carboxylic acid), a precursor of ethylene, down-regulated *miR159*, *miR164*, *miR319*, *miR390* and *miR396* in roots of *Medicago truncatula* [43]. Except for *miR159* and *miR319*, the expression of *miR164*, *miR390* and *miR396* were also repressed by ethylene in rose petals. Further studies are needed to investigate whether *miR159* and *miR319* were regulated by ethylene in an organ-specific manner.

### Ethylene-Responsive miRNA/mRNA Modules in Rose Petals

Integrated analysis of expression of miRNAs and their targets can help to identify miRNA/mRNA modules involved in regulating specific biological processes, such as cold stress in wheat [44].

During the past decade, genomics approaches have been applied in rose and several EST libraries from rose petals were reported [45], [46], [47]. In 2008,∼10,000 ESTs were deposited in public databases, including GenBank and GDR (Genome database for the Rosaceae, http://www.rosaceae.org/). Based on these data, expression profiles of 4,765 transcripts were detected in roses from floral transition to flower senescence using a newly developed Affymetrix microarray [48]. Interestingly, we found that several genes, whose expression was changed during the early flower opening period, were potential targets of miRNAs, such as expansin (*miR5139*), MYB (*miR159*), and NAC (*miR164*). Functional analysis of miRNAs in the early flower opening period will be helpful for understanding this process.

Moreover, application of next-generation sequencing technologies greatly promoted the study on rose genomics. Kim et al reported a rose transcriptome database, which contained more than 30,000 transcripts. According to target prediction and gene expression analysis, several conserved miRNAs, such as *miR156*, *miR159*, and *miR396*, were proposed to be involved in regulating genes related to coloring, including those in the flavonoid biosynthetic pathway [28]. Lately, a transcriptome dataset containing 80,714 transcript clusters was generated by using the RNA from various tissues of *R. chinensis* cv. ‘Old Blush’ and in response to biotic and abiotic stresses [49].

We also constructed a floral transcriptome database containing 60,944 transcripts assembled from transcriptome sequences generated using the 454 sequencing technology (http://bioinfo.bti.cornell.edu/rose). Furthermore, based on microarray analysis, we identified 2,189 unique ethylene-responsive transcripts. In the present study, these transcripts were used to identify the ethylene-responsive miRNA/target modules. We were able to identify a total of seven ethylene-responsive miRNA/mRNA modules (Table 7). Quantitative RT-PCR confirmed that all identified miRNA/target modules exhibited negatively correlated expression profiles between miRNAs and their corresponding targets. Except *miR164*, all miRNAs have not been reported to be ethylene-responsive. Interestingly, the identified modules included well-conserved miRNAs (*miR156*, *miR164* and *miR166*), a less-conserved miRNA (*miR5139*), and a novel miRNA (*rhy-miRC1*), suggesting profound and broad impacts of ethylene on plant development.

In the identified modules, targets of *miR164* (NAC) and *miR156* (SPL) are transcription factors. In *Arabidopsis thaliana*, *miR164* regulates *NAC1* and several *NAC* genes of the NAM subfamily. Consequently, it regulates many aspects of plant development. For instance, *miR164* was found to be regulated by developmental cues and control organ boundary maintenance and leaf development [50]–[52], while it was also found to be auxin-responsive and regulate *NAC1* to control lateral root initiation [53]. Recently, *miR164* was found to be ethylene-responsive and regulated leaf senescence in *Arabidopsis thaliana* [54] and cell expansion in rose petals [Pei et al., unpublished data].

Like *miR164*, *miR156* is also a well conserved miRNA in plants. The *miR156/SPL* module was previously reported to directly regulate *FLOWERING LOCUS T* (*FT*) expression to control ambient temperature-responsive flowering. A recent report showed that a *miR156*-targeted SPL protein could destabilize a MYB-bHLH-WD40 transcriptional activation complex to influence expression of anthocyanin biosynthetic genes in *Arabidopsis thaliana* [55]. High level of *miR156* decreased the accumulation of anthocyanins, while low *miR156* activity caused high levels of flavonols. Interestingly, ethylene increased expression levels of chalcone synthase (CHS), flavanone 3-hydroxylase (F3H), and UDP glucose-flavonoid 3-O-glucosyl transferase (UFGT), and consequently promoted the accumulation of anthocyanins in the skin of grape berries [56]. Since ethylene also significantly increased *miR156* abundance in rose petals, whether ethylene regulates anthocyanin accumulation in rose petals through modulating the *miR156/SPL* module is worthy of future investigation.

In addition, *miR166* and *miR5139* appeared to target beta-galactosidase and expansin genes, respectively, in an ethylene-regulated manner. Beta-galactosidase and expansin genes are involved in cell-wall modification [57], [58], which has been proven to be very sensitive to ethylene treatment in *Arabidopsis thaliana* and tomato [59]–[61]. Whether miRNAs, such as *miR166* and *miR5139*, are involved in ethylene-regulated cell wall modification in rose petals needs further investigation. Intriguingly, we identified an ethylene responsive module that included a novel miRNA, *rhy-miRC1*, and its target RU25062 that showed no homology to any known genes, indicating the function of *rhy-miRC1*/RU25062 might be specific to rose.

### Conclusions

Here, we reported a set of miRNAs identified from rose (*Rosa* sp.) petals during early flower opening and in response to ethylene treatment. We found that expression of 28 known and 39 novel miRNAs was changed in rose petals during early opening process, and 28 known miRNAs and 22 novel miRNAs were responsive to ethylene treatment. Furthermore, integrated analysis of expression profiles of miRNAs and their targeted mRNAs in response to ethylene, an important factor influenced flower opening and senescence in rose, identified seven miRNA/mRNA modules. These modules might be important downstream regulatory components which facilitate the function of ethylene in flower opening, senescence, or both.

## Materials and Methods

### Flower Treatment, RNA Isolation, and Small RNA Sequencing

Flowers of cut roses (*Rosa hybrida*) cv Samantha were harvested at stage 0 and 2 of flower opening [25], respectively (We state clearly that no specific permissions were required for these locations/activities and confirm that the field studies did not involve endangered or protected species). The flowers were immediately put into tap water after harvest and then transported to the laboratory within 1 h. For ethylene treatment, stems of stage 2 flowers were cut to 25 cm under water, and then placed in deionized water (DW). The flowers were treated by 10 ppm ethylene in a sealed airtight chamber for 24 h, and flowers exposed to air were used as the control. Treatments were conducted at 23–25°C and 1 mol L^−1^ NaOH was placed in the chamber to prevent the accumulation of CO_2_ [62]. The samples were immediately collected after treatment. The 2^nd^ and 3^rd^ whorl petals of more than 15 flowers were collected and pooled together. Total RNAs were isolated using the pBiozol reagent (BioFlux, Hangzhou, China). Small RNA libraries were prepared according to the manufacturer's instructions and sequenced on an Illumina HiSeq2000 system.

### Bioinformatics Analysis of sRNA Sequences

The raw sequencing data were processed to trim the adapter sequences and remove low quality sequences, and rRNA and tRNA sequences were also removed. The cleaned small RNA sequences with expression>5 RPM (reads per million) in at least one of the four libraries were aligned to the strawberry genome [33] and the rose transcriptome database (http://bioinfo.bti.cornell.edu/rose) using Bowtie [63] with perfect matches. Only sRNAs with no more than 20 hits were kept and their flanking sequences on the genome or transcriptome (200 bp on each side) were extracted and then folded in silico using the RNAfold program [64]. Resulting folded structures were checked with miRcheck [65] with default parameters. Candidate miRNAs whose precursors passed miRcheck were then aligned to the miRNA database, miRBase 17.0, using Bowtie [63] allowing up to 2 mismatches. The miRNAs shared homology to known miRNAs were identified as conserved miRNA candidates. Then, they were further confirmed by checking their corresponding precursor structures. Only the candidates with expected structures were identified as conserved miRNAs.

After identifying all candidate miRNAs, those which did not share homology to all known sequences in miRBase were regarded as novel miRNA candidates. And the novel miRNAs' precursor structures were further analyzed by miRcheck [66]. Potential miRNA star sequences were identified from the sRNA dataset to provide additional evidence supporting miRNA predictions. For novel miRNA candidates without corresponding miRNA star sequences identified, only those expressed in all four samples were kept. MiRNA targets were identified according to the scoring matrix described previously [66]. Briefly, all of the conserved and novel rose miRNAs were aligned against rose transcriptome dataset (http://bioinfo.bti.cornell.edu/rose) using a BLASTn search strategy. For evaluation of the complementary sites between predicted rose miRNAs and potential mRNA targets, no more than four mismatches between miRNAs and their potential mRNA targets (G:U was regarded as 0.5 mismatch), and no mismatch between positions 10 and 11.

### Quantitative RT-PCR

The stem-loop RT-PCR was performed as described previously [67]. For each miRNA, 1 µg DNase I-treated total RNA was used in the reverse transcription reaction with SuperScript III (Invitrogen). 5S rRNA was used as the internal control. For quantitative RT-PCR of mRNAs, 1 µg DNase I-treated total RNA was used to synthesize cDNA by M-MLV (Promega) using poly(dT)^18^ oligonucleotides. *RhACT5* was used as the internal control. SYBR Green PCR Master Mix (Applied Biosystems) was used in all quantitative RT-PCR experiments. The relative expression changes of miRNAs and genes were calculated using the 2 d-d Ct method [68]. Primers used in all quantitative RT-PCR experiments are listed in Table S8.

### Plasmid Construction and Transformation

To construct sensor plasmid, the putative miRNA target site sequence was fused to the 5′-end of *EGFP* in frame. The resulted fusion sequences were inserted into a modified binary vector pCAMBIA 1300 harboring a Super promoter. The resulting sensor constructs were transformed into *Agrobacterium* strain GV3101 and then used to co-infiltrate the tobacco leaves with plasmid harboring corresponding miRNA foldbacks. After 3 days of co-infiltration, tobacco leaves were harvested and used to extract total RNA for RLM-RACE analysis.

### RLM-RACE

The 5′ RLM-RACE was carried out using the FirstChoice RLM-RACE Kit (Ambion). Two microgram total RNA was directly ligated to 5′ RACE oligo adaptor without calf intestine alkaline phosphatase and tobacco acid pyrophosphatase treatments. The ligated RNA was used to synthesize the cDNA. The PCR products were gel-purified and cloned into the pGEM-T Easy vector (Premega, Madison, WI, USA), and randomly selected clones were sequenced. For the RU15050 and RU13577 sensor sequences, a set of general primers designed based on the *EGFP* sequence were used. For RLM-RACE using total RNA from rose petals, gene-specific primers were used. All primers were listed in Table S8.

## Supporting Information

Figure S1 **Digital expression profiles of** ***miR171*** **,** ***miR171*** *, ***miR396*** **and** ***miR396*** * in rose petals during earlier opening period and in response to ethylene.
(DOC)

Table S1 **The sequencing results of small RNAs from 4 rose flower samples.** The flowers of stage 2 were treated by 10 ppm ethylene in a sealed airtight chamber for 24 h, and flowers exposed to air were used as the control.
(XLS)

Table S2 **Prediction of known miRNA precusors.** The rose floral transcriptome database, and the genome sequence of strawberry (F. vesca) and transcriptome data of rose were used as reference, respectively. R, rose; S, strawberry.
(XLS)

Table S3 **Prediction of novel miRNA and their precusors.** The rose floral transcriptome database, and the genome sequence of strawberry (F.vesca) were used as reference, respectively. R, roses; S, strawberry.
(XLS)

Table S4 **Reads of known and novel miRNAs in rose petals.** For each library, petals from 15 flowers were mixed and used to avoid the individual difference. The miRNAs in bold indicate highly expressed miRNA in petals (more than 50 RPM in at least one library).
(XLS)

Table S5 **Predicted targets of known miRNAs in roses.**
(XLS)

Table S6 **Predicted targets of novel miRNAs in roses.**
(XLS)

Table S7 **Integraded analysis of expression profiles of miRNAs and responsed targets in rose petals.**
(XLS)

Table S8 **Oligonucleotide primer sequences.**
(XLS)

Table S9 **Comparison of known and novel miRNAs identified by Kim et al and in our study.**
(XLSX)
