# Supplementary material for: Integrative Analysis of miRNA and mRNA Profiles in Response to Ethylene in Rose Petals during Flower Opening
Source: PLoS One. 2013 May 16;8(5):e64290. doi: 10.1371/journal.pone.0064290 (PMC3655976; doi:10.1371/journal.pone.0064290)
Supplement: Figure S1 — Digital expression profiles of miR171 , miR171 *, miR396 and miR396 * in rose petals during earlier opening period and in response to ethylene. (DOC) [file pone.0064290.s001.doc]

# Supplemental Figures


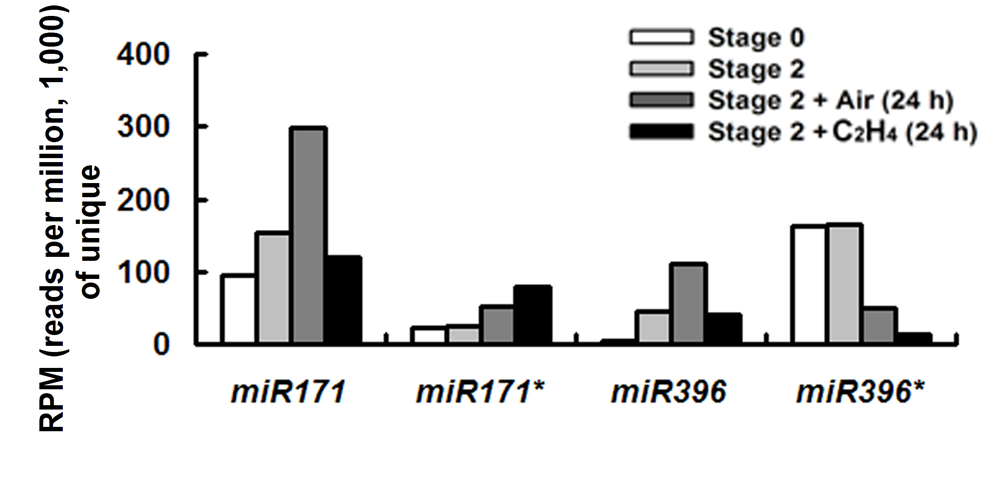


**Figure S1. Digital expression profiles of *miR171*, *miR171**, *miR396* and *miR396** in rose petals during earlier opening period and in response to ethylene.**
